# Divergent roles of red cell arginase in humans and mice: RBC Arg1 KO mice show preserved systemic l-arginine bioavailability and infarct size *in vivo*

**DOI:** 10.1016/j.redox.2025.103768

**Published:** 2025-07-14

**Authors:** Sophia K. Heuser, Junjie Li, Zhixin Li, Anthea LoBue, Kyle Heard, Julia Hocks, Tatsiana Suvorava, Ron-Patrick Cadeddu, Corinna Strupp, Luke Dunaway, Zhengbing Zhuge, Stacy L. Gelhaus, André Heinen, Ulrich Germing, Martin Feelisch, Mattias Carlström, Brant Isakson, Malte Kelm, Jon O. Lundberg, Miriam M. Cortese-Krott

**Affiliations:** a Myocardial Infarction Research Laboratory, Department of Cardiology, Pulmonology, and Angiology, Medical Faculty, Heinrich-Heine-University, Germany; b Department of Hematology, Oncology and Clinical Immunology, Heinrich-Heine-University, 40204, Düsseldorf, Germany; c Robert M. Berne Cardiovascular Research Center, University of Virginia School of Medicine, Charlottesville, VA, USA; d Department of Physiology and Pharmacology, Karolinska Institute, Stockholm, Sweden; e Department of Pharmacology and Chemical Biology, University of Pittsburgh School of Medicine, Pittsburgh, PA, USA; f Department of Physiology, Medical Faculty, Heinrich-Heine-University, Germany; g Clinical & Experimental Sciences, Faculty of Medicine, University of Southampton, Southampton, United Kingdom; h Clinic of Cardiology, Pulmonology and Angiology, Medical Faculty, Heinrich Heine University of Düsseldorf, Düsseldorf, Germany; i CARID, Cardiovascular Research Institute Düsseldorf, Medical Faculty, Heinrich-Heine-University, Düsseldorf, Germany

**Keywords:** Cre/LoxP system, RBC-specific Arg1, NO bioavailability, Systemic l-arginine metabolism, Erythroid cell differentiation, Acute myocardial infarction, Erythrocytes

## Abstract

In humans and other primates, red blood cells (RBCs) constitutively express high levels of liver-type arginase 1 (Arg1), which regulates systemic l-arginine and nitric oxide (NO) bioavailability, particularly under pathological conditions such as sickle cell disease. In contrast, the role of RBC Arg1 in mice *in vivo* remains poorly defined. Here, we investigated the contribution of RBC Arg1 to systemic l-arginine metabolism, NO bioavailability, and cardioprotection following acute myocardial infarction *in vivo*. Comparative analyses of human blood fractions revealed that arginase activity in RBCs is comparable to that in white blood cells and is predominantly localized to the RBC membrane. In contrast, arginase activity in mouse RBC membranes was 13,500-fold lower as compared to human RBC membranes as measured by ^13^C-l-ornithine formation. To assess the *in vivo* relevance of RBC Arg1, we generated RBC-specific Arg1 knockout (KO) mice using the Cre/loxP technology. RBC Arg1 KO mice exhibited normal erythropoiesis and hematologic parameters. Moreover, systemic l-arginine and l-citrulline levels were preserved, while l-ornithine levels were lower in plasma of RBC Arg1 KO mice as compared to wildtype controls; whereas circulating NO metabolites, systemic hemodynamics, cardiac function, and infarct size post-acute myocardial infarction were preserved. These findings demonstrate that, unlike in humans, in mice RBC Arg1 plays a negligible role in regulating systemic l-arginine homeostasis and cardioprotection, underscoring critical interspecies differences and the need for human studies to evaluate the pathophysiological relevance of RBC arginase.

## Highlights

• In human blood WBCs and RBCs show comparable arginase activity.
• Mouse RBC Arg1 activity is significantly lower compared to human RBCs.
• In mice, WBCs exhibit the highest Arg1 activity among blood cells.
• RBC Arg1 KO mice show preserved l-arginine and NO bioavailability.
• RBC Arg1 KO mice show preserved systemic hemodynamics and infarct size post-AMI.

## 1 Introduction

Arginase (E.C. 3.5.3.1) is a key enzyme of the urea cycle, catalyzing the conversion of l-arginine into l-ornithine and urea, and is abundantly expressed in the liver [1]. There are two different isoforms of arginase, arginase 1 (Arg1) and arginase 2 (Arg2), which differ in tissue expressional level, intracellular localization, and cell/organ-specific function [[1], [2], [3], [4]]. Arg1 is mainly expressed constitutively in the cytoplasm of hepatocytes, while Arg2 is a mitochondrial enzyme, highly abundant in the kidney. In non-hepatic cells, Arg1 and Arg2 were initially proposed to regulate l-arginine bioavailability and control nitric oxide (NO) synthesis by limiting substrate availability for nitric oxide synthase (NOS) enzymes [[5], [6], [7], [8], [9], [10], [11]]. However, these effects likely occur only when arginase expression is upregulated under pro-inflammatory and pathological conditions, and in aging blood vessels [1,5,[12], [13], [14], [15], [16]]. Another function proposed for Arg1 and 2 is the synthesis of l-ornithine as a precursor of polyamines like spermidine and spermine, which control cell proliferation and survival [[17], [18], [19], [20]]. From an evolutionary perspective, arginases are more ancient than NOS enzymes, suggesting that regulation of polyamine synthesis represents a primordial function of arginases [21,22].

Genetic defects of Arg1 in humans and other primates cause hyperarginemia, which leads to progressive spastic diplegia or quadriplegia, intellectual disability, seizures, and growth retardation [[23], [24], [25], [26], [27], [28], [29], [30]]. Mouse models of inducible or liver-specific Arg1 knock outs (KOs) show also hyperarginemia, while the constitutive whole body Arg1 KO mice die 2 weeks after birth due to hyperammonemia and hyperarginemia [[31], [32], [33]], as recently reviewed [1].

Human red blood cells (RBCs) exhibit high expression and activity of Arg1 [[34], [35], [36], [37], [38], [39], [40], [41], [42]]. Accumulating evidence indicates that activity of red cell Arg1 may contribute to regulate l-arginine bioavailability. Circulating RBCs from patients and primates with genetic defects of Arg1 and hyperarginemia lack arginase activity also in RBCs [28,37,38,43]. On the other hand, an increase in RBC arginase activity has been reported in hematological diseases such as irondeficiency anemia, megaloblastic anemia, sickle cell disease and hemolytic uremic syndrome [34,[44], [45], [46]]. Consumption of l-arginine by red cell Arg1 released into plasma during hemolysis in sickle cell disease and hemolytic uremic syndrome has been correlated to decreased arginine bioavailability and endothelial dysfunction [45,46].

Interestingly, Arg1 expression in RBCs is very high in humans, gorillas, chimpanzees, and other higher primates [35]. In contrast, rat and mouse RBCs are known to express much lower levels of Arg1 [35,41,47]; and its role *in vivo* remains poorly defined. The aim of this study was to investigate the specific role of RBC Arg1 in regulating systemic l-arginine metabolism, NO bioavailability, and cardioprotection following acute myocardial infarction (AMI) *in vivo*. To this end we compared arginase activity across blood cell subpopulations in humans and mice and generated RBC-specific Arg1 KO mice (RBC Arg1 KO). We find that in mice, in contrast to humans, RBC Arg1 plays a negligible role in regulating systemic l-arginine homeostasis, NO metabolism and cardioprotection, underscoring critical interspecies differences and the need for human studies to evaluate the pathophysiological relevance of RBC Arg1.

## 2 Material and methods

### 2.1 Chemicals

Unless otherwise specified, chemicals and labelled internal standards were purchased from Sigma-Aldrich (Darmstadt, Germany).

### 2.2 Collection of human blood samples

Venous blood was collected from five healthy volunteers after obtaining written informed consent. All procedures were conducted in compliance with the Declaration of Helsinki and approved by the ethics committee of the Heinrich-Heine-University (Study number 5788R). Blood was collected in BD vacutainers and anticoagulated with EDTA. Blood centrifugation and the RBC membrane (ghost) preparation were conducted using the same procedures as described for the mouse blood, as outlined in sections 2.9, 2.10 below.

### 2.3 Animals

All animal experimental protocols were approved by the LANUV (Landesamt für Natur, Umwelt und Verbraucherschutz Nordrhein-Westfalen, Germany) and the regional Institutional Animal Care and Use committee from Karolinska Institute Sweden. Animal care followed institutional guidelines from HHU or Karolinska Institutet. Arg1^flox/flox^ mice were purchased from the Jackson Laboratory (Jax stock: 008817) [48]. RBC-specific Arg1 KO mice were generated by crossing homozygous Arg1^flox/flox^ mice with erythroid-specific Hbb-Cre^pos^ mice [49] to obtain RBC Arg1^flox/flox^ Hbb-Cre^pos^ and Arg1^flox/flox^ Hbb-Cre^neg^ mice (WT). Male mice of 3–6 months of age were used. Experimental groups were randomized based on genotype. Genotyping was carried out by Transnetyx (Cordova, Tennessee, USA).

### 2.4 Blood collection, blood count, and reticulocyte count

Mice were treated with buprenorphine (0.1 mg/kg), anesthetized with isoflurane (3.0 % induction and 2.5 % maintenance) and killed by exsanguination by rapidly collecting large amount of blood (ca. 1 mL) by cardiac puncture and anticoagulated with 5 mM EDTA. Blood cell count was performed using a Coulter counter. Reticulocytes were enumerated by flow cytometry essentially as described. Briefly, 2 μL of whole blood was added to 2 mL staining solution containing 0.1 μM thiazole orange in phosphate buffered solution (PBS), and incubated for 30 min at room temperature. For each sample, an unstained control was prepared by diluting 2 μL of blood in PBS to determine the background intracellular fluorescence. Reticulocytes percentage was calculated as the percentage of stained cells in the total RBC population.

### 2.5 Bone marrow cells collection

Bone marrow was isolated from the tibia and femur (four bones per mouse) by cutting both bone ends with scissors, placing the bones in a 0.5 mL tube with a perforated bottom, which was nested in a 2 mL collection tube, and centrifuged at 10,000×*g* for 30 s at 4 °C.

### 2.6 Analysis of DNA recombination in the bone marrow

The Cre recombinase-dependent genetic recombination of the DNA locus was determined in the bone marrow by real-time polymerase chain reaction (real-time PCR) with specific Taqman primers and probes, which were designed to recognize the floxed allele (Arg1^flox/flox^) and the allele carrying the genetic deletion (ΔArg1) (Table S1). Primers and probes were designed by Transnetyx (Cordova, Tennessee, USA).

### 2.7 Analysis of terminal erythroid precursors in bone marrow and spleen

Single-cell suspensions of bone marrow cells were obtained by resuspending bone marrow in PBS and by filtering it through a 40 μm cell strainer. Single-cell suspensions from mouse spleen were obtained by pushing the spleen through a 40 μm filter by using a syringe plunger and then washing the filter with PBS. Erythroid terminal differentiation in these cell preparations was determined in the CD45^−^ cell fraction by flow cytometry as described [50]. Briefly, cell fractionation was carried out by the depletion of CD45^+^ cells from single-cell suspension obtained from bone marrow or spleen by magnetic separation using anti-CD45 microbeads and LS columns (Miltenyi Biotech, Bergisch-Gladbach, Germany). The CD45^−^ cells collected in the flow through were pelleted by centrifugation at 500×*g* for 10 min at 4 °C, resuspended in 2 mM EDTA 0.5 % bovine serum albumin (BSA) in PBS and then stained with anti-CD45, anti-CD44 and anti-Ter119 antibodies together with 7-AAD as viability staining. Dead cells and CD45^+^ cells were gated out before subpopulation analysis. The proerythroblast population was gated as CD44^hi^/Ter119^low^. Basophilic, polychromatic, orthochromatic erythroblasts, reticulocytes, and RBCs were gated by size (using the foreword scatter, FSC) and by CD44 expression.

### 2.8 Isolation of erythroid cells from bone marrow

Erythroid cells (Ter119^+^ CD45^−^) were isolated from the bone marrow of RBC Arg1 KO mice and WT mice by magnetic microbeads-assisted cell separation (MACS) according to the manufacturer's protocols (Miltenyi Biotec, Bergisch Gladbach, Germany) as described in detail elsewhere [51]. In brief, the extraction was performed by two independent steps of magnetic separation consisting of a negative selection with anti-CD45 microbeads and a positive selection with anti-Ter119 microbeads. The purity and yield of cells were determined by flow cytometry analysis using specific antibodies anti-CD45 and Ter119 according to standard procedures. After extraction, Ter119^+^ cells were preserved in RNA-later (Merck, Darmstadt, Germany) and kept at −80 °C until later use.

### 2.9 Isolation of platelets, white blood cells and RBCs

**Fig. 1:**
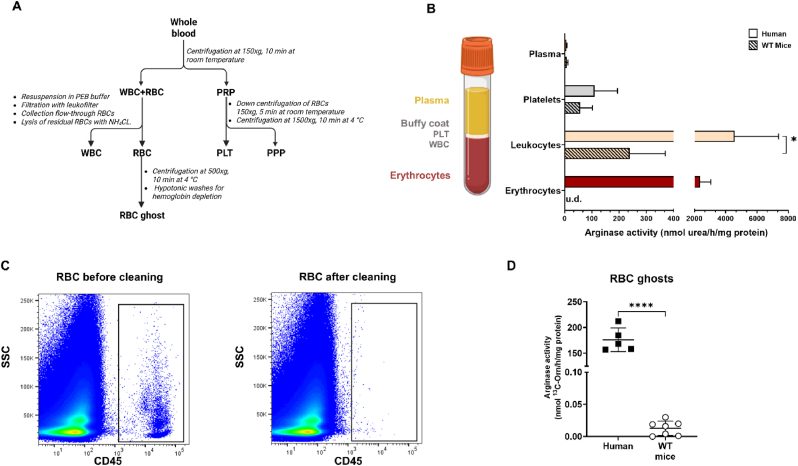
**Analysis of arginase activity in blood cell subpopulations from human and mouse blood. (A)** Whole blood was collected, anticoagulated with 5 mM EDTA, and centrifuged at low speed to separate platelet rich plasma (PRP) from WBCs and RBCs. PRP underwent another centrifugation step at higher speed to separate platelets (PLTs) and platelet poor plasma (PPP). WBCs and RBCs were separated by filtration with WBC-specific membrane (leukofiltration) and collected by centrifugation; RBC membranes (ghosts) were prepared from RBC pellets by 3–4 consecutive treatments with cold hypotonic buffer (PBS diluted 1:27 with MilliQ water pH = 7.4) at 0 °C. **(B)** Comparison of arginase activity determined colorimetrically as production of nmol urea/h/mg proteins in different blood cellular fractions collected from human healthy volunteers and WT mice. (Welch's *t*-test ∗p < 0.05.) **(C)** Purity of RBC suspension was tested by flow cytometric analysis before and after filtration through a leukodepletion filter. The presence of CD45^+^ cells (leukocytes) is shown on the left panel and their significant depletion is shown in the right panel. **(D)** Comparison of arginase activity in RBC membrane preparations (ghosts) obtained from human blood and WT mouse blood determined by mass spectrometry as production of ^13^C-L-ornitine nmol/h/mg protein (Welch's *t*-test, ∗∗∗∗p < 0.0001).

For the isolation of RBC from other blood cells, an optimized protocol using centrifugation steps and leukofiltration, was carried out (see scheme in Fig. 1A). Whole blood was centrifuged at 150×*g* for 10 min at room temperature to separate platelet rich plasma (PRP) from white blood cells (WBCs) and RBCs. PRP was further cleaned up by a second centrifugation at 150×*g* for 5 min at room temperature and subjected to centrifugation at 1500×*g* for 10 min at 4 °C, which separates platelets from platelet poor plasma (PPP). Both fractions were collected and stored at −80 °C until use. The pellet, containing WBCs and RBCs, was resuspended to the starting blood volume (800–1000 μL) in a cell separation buffer containing 0.5 % BSA, 2 mM EDTA in PBS at pH 7.5. Leukofilters (Acrodisc, Pall, City, Country) were mounted on a 5 mL syringe, equilibrated with the separation buffer and then the cell suspension was applied to the filters, and filtered through by gravity; the filters were washed with 2 mL separation buffer for 5 consecutive times. The RBCs in the eluate were centrifugated at 500×*g* for 10 min at 4 °C, the supernatant discarded, and the pellet used for preparation of RBC ghosts. WBCs were washed out of the filter by flipping the filter and flushing it with 1 mL PBS using the syringe plunge and then pelleted at 500×*g* for 10 min at 4 °C and the supernatant discarded. Residues of RBCs were removed by lysis with 1 mL cold ammoniochloride buffer (0.8 % NH_4_Cl, 0.1 % NaHCO_3_, 10 mM EDTA, pH 7.4) on ice for 10 min. WBC were snap frozen in liquid N_2_ and stored at −80 °C.

### 2.10 Preparation of RBC ghosts

RBC membrane preparations or “ghosts” were prepared by cold hypotonic permeabilization of RBC membranes and removal of intracellular hemoglobin. Briefly, leukodepleted RBC pellets obtained from ca. 800 μL blood were resuspended in 800 μl of a freshly prepared cold (4 °C) hypotonic buffer (1:27 dilution of PBS in cold water, pH = 7.4) and incubated on an ice/ethanol bath at 0 °C for 30 min followed by centrifugation at 16,000×*g* at 4 °C for 20 min. The pellets were resuspended in the initial volume of hypotonic buffer, and the incubation/centrifugation steps were repeated 3 to 6 times until white ghosts were obtained.

### 2.11 Analysis of Arg1 expression by real-time RT-PCR

Gene expression of Arg*1* mRNA in targeted and non-targeted tissues and cells was determined by real-time reverse transcription (RT)- PCR according to protocols described before [51]. Briefly, cells and organs were homogenized in RNA lysis buffer containing 2-mercaptoethanol by using the Tissue Ruptor (Qiagen, Hilden, Germany). The tissue lysates from fibrous tissues (aorta, heart) were treated with 10 μL proteinase K (Qiagen, Hilden, Germany) for 10 min. Total RNA was extracted by using RNeasy Mini Kit (Qiagen, Hilden; Germany) combined with DNase I digestion following the manufacturer's instructions. The concentration of total RNA was measured by using the NanoDrop One spectrophotometer (Thermo Fisher Scientific, Schwere, Germany). RT reactions of up to 1 μg RNA were performed using QuantiTect reverse transcription kit (Qiagen, Hilden, Germany). Due to the low concentration of RNA in Ter119^+^ cells lysate, the samples were pre-amplified using SsoAdvanced PreAmp Supermix (BioRad, Düsseldorf, Germany). Analysis of mRNA expression was carried out by real-time PCR using TaqMan primers and probes together with the Fast Advanced Master Mix (Thermo Fisher Scientific, Schwerte, Germany) in the Applied Biosystems StepOnePlus Real-time PCR System. Data for mRNA expression were analyzed by using the ΔΔCT method as described previously [52].

### 2.12 Analysis of Arg1 expression by western blotting

RBC membrane preparations (“ghosts”) and organs from WT and RBC Arg1 KO mice were lysed in radioimmunoprecipitation assay (RIPA) buffer (0.5 % sodium deoxycholate, 0.1 % SDS, and 1 % NP40 in PBS, pH 7.4) with EDTA-free protease inhibitor cocktail (Roche, cat. No. 11836170001). The samples were homogenized on ice by using Tissue Ruptor (Qiagen, Hilden, Germany), sonicated for 1 min, then cleared by centrifugation at 10,000×*g* for 10 min at 4 °C. The total protein concentration of the supernatant was determined by using the Lowry assay (BioRad, Düsseldorf, Germany). Lysates were loaded on 10 % SDS-polyacrylamide gels prepared by using ready polyacrylamide (Carl Roth, Karlsruhe, Germany) and transferred onto nitrocellulose membrane (Amersham Biosciences, Munich, Germany). Membranes were then blocked for 1 h with 5 % low-fat milk in T-TBS (10 mM Tris, 100 mM NaCl, 0.1 % Tween). After the blocking step, membranes were incubated overnight at 4 °C with a mouse anti-Arg1 (1:50 in T-TBS) (BD, Bioscience, BD transduction laboratories cat. 610708), mouse anti-beta actin (1:1000 diluted in T-TBS) (Sigma cat. A1978-100UL), or mouse anti-GAPDH (glyceraldehyde-3-phosphate dehydrogenase) (1:1000 in T-TBS) (Sigma cat. G8795-100UL) antibody. Membranes were washed multiple times for a total of 30 min with T-TBS, incubated with a secondary antibody HRP goat anti-mouse antibody (1:5000) in 5 % milk (BD Pharmingen, cat.554002) for 1 h at room temperature, and then washed multiple times for a total of 30 min in T-TBS at room temperature. The bands were detected by using West Pico or West Femto Chemiluminescence Detection Reagent (ThermoFisher, Darmstadt, Germany) in Invitrogen iBright FL1000 (ThermoFisher, Darmstadt, Germany). The bands were quantified by using Image J (Rasband, W.S., ImageJ, U. S. National Institutes of Health, Bethesda, Maryland, USA, https://imagej.nih.gov/ij/, 1997–2018).

### 2.13 Determination of Agr1 expression by immunotransmission electronic microscopy

The immunotransmission electronic microscopy (TEM) was performed a previously described [53]. RBCs were isolated by cardiac punction and fixed in 4 % PFA containing 0.05 % glutaraldehyde. RBCs were spun down, embedded in LR White, and sectioned into 70 mm sections. RBC were stained overnight with 1:10 rabbit anti-Arg1 antibody. Goat anti-rabbit 12 nm gold beads (1:50) were used to resolve protein localization on the RBCs and imaged using an electron microscope.

### 2.14 Analysis of arginase activity

Arginase activity was measured in RBC lysates, RBC ghosts, PLT, WBC, plasma and organ lysates by assessing the enzymatic synthesis of urea from l-arginine by derivatization with α-isonitrosopropiophenone [41], a method originally described by Archibald [54,55]. See also [1] for an overview and discussion of the methods. Incubation time and amount of protein used in the assay was carefully optimized in preliminary experiments, as described in Table S2. Samples were diluted to a defined total amount of protein (2 μg–250 μg protein) depending on the tissue/organ (please refer to Table S2) in 50 mM Tris buffer at pH 7.4 and divided in two aliquots, one of each was heat inactivated at 100 °C for 10 min and cooled on ice. Both aliquots were then pre-treated with 10 mM MnCl_2_ and incubated for 10min at 60 °C to activate arginase. Afterward, l-arginine (500 mM in 50 mM Tris pH 9.7) was added and incubated for 15 min to 5 hours (h) at 37 °C, depending on the tissue/organ, please refer to Table S2. The reaction was stopped by using an acid mix (H_2_SO_4_, H_3_PO_4_, and H_2_O, 1:3:7 in volume ratio). To detect the urea concentration, samples and standards were treated with 9 % α-isonitrosopropiophenone (final dilution 1:24, final concentration 0.375 %) and incubated for 1 h at 100 °C. The urea concentration was determined by measuring the absorbance at 540 nm. The heat-inactivated aliquot from each sample was used as the corresponding background control. The background corrected signal was used to calculate arginase activity in nmol urea/h/mg protein.

### 2.15 Analysis of arginine bioavailability and arginase activity by LC-MS

The concentrations of l-arginine, l-ornithine, and l-citrulline in plasma were detected by LC-MS as described before [13]. Plasma was diluted 1:10 with cold 0.1 % formic acid in MeOH for protein precipitation and centrifuged for 10 min at 16,000×*g*. The supernatant was spiked with 2 μM ^13^C-labelled l-arginine (internal standard) for further quantification. Arginase activity in RBC ghosts was also assessed by measuring the formation of ^13^C-l-ornithine from ^13^C-l-arginine. Samples were diluted to a total protein amount of 250 μg in 100 μL of 50 mM Tris buffer (pH 7.4), pre-treated with 125 μL of 10 mM MnCl_2_, and incubated for 10 min at 60 °C to activate arginase. Subsequently, 25 μL of ^13^C-l-arginine (100 mM in 50 mM Tris, pH 9.7) was added, followed by incubation for 3 h at 37 °C. Ten volumes of ice-cold MeOH (with 0.1 % formic acid) were then added to stop the reaction, and the mixture was centrifuged at 16,000×*g* for 10 min at 4 °C. The supernatants were transferred to clean Eppendorf tubes and dried using vacuum centrifugation (SpeedVac). Samples were then resuspended in 200 μL of MeOH (with 0.1 % formic acid) and loaded onto an Agilent 1290 Infinity HPLC system coupled to a 6550 QTOF-MS (Agilent Technologies, Waldbronn, Germany), equipped with an electrospray ionization source. Standards or samples (10 μL) were injected into the LC system separated on an Agilent ZORBAX RRHD HILIC Plus column (1.8 μm, 2.1 mm × 100 mm, with a 1.8 μm, 2.1 mm × 5 mm guard column) by a gradient formed by mixing 0.1 % formic acid in water (A) and 0.1 % formic acid in acetonitrile (B) as follows: 3 % A (0 min), 10 % (1 min), 45 % (4 min), held to 5 min, then down to 3 % by 6 min and re-equilibrated to 10 min. MS detection was in positive mode (scan 100–1700 *m*/*z*), 200 °C source, 12 L/min drying gas, 35 psig nebulizer, 330 °C sheath gas at 11 L/min, 3000 V and 125 V for capillary and fragmentor voltage, respectively.

### 2.16 Measurement of erythropoietin, transferrin, ferritin in plasma

Concentrations of transferrin (Abcam, ab157724), ferritin (Abcam, ab157713), and erythropoietin (ThermoScientific, EN28RB) levels in plasma were quantified by ELISA according to the manufacture's instruction. Absorbance was measured with a FLUOstar Optima (BMG Labtech).

### 2.17 Determination of NO metabolites in blood and tissues

Nitrite, nitrate, nitrosyl heme (NO-heme), and nitrosated (S-nitroso and N-nitroso) products (RXNO) were quantified in organs, plasma, and RBCs using HPLC (ENO-30, Eicom) and ozone-based gas-phase chemiluminescence (nCLD 88, Eco Physics), respectively, as previously described [56].

### 2.18 Measurement of flow-mediated dilation by ultrasound

Vascular function was measured as flow-mediated dilation (FMD) and assessed with a Vevo 2100 with a 30–70 MHz linear array microscan transducer (VisualSonics) as described [57]. Briefly, we measured the changes in vessel diameter of the iliac artery in response to shear stress, following release of a 5 min vascular occlusion achieved by inflating an intravascular cuff to suprasystolic pressure levels. During the experiment, mice were kept under anesthesia of 1.5–2 % isoflurane, with heart rate ranging 400 to 500 bpm, a breathing rate of about 100 breaths/min and body temperature of 37 °C.

### 2.19 Determination of vascular reactivity *ex vivo* by vessel myography

Functional studies for conductance vessels were carried out as described [58,59]. In brief, aorta was immediately excised following euthanasia and transferred in ice-cold physiological saline solution (PSS) until use. Vessel rings (2 mm) were mounted to a myograph chamber (model 620 M; Danish MyoTechnology, Denmark), and thereafter allowed to equilibrate for 45 min in PSS bubbled with carbogen gas (95 % oxygen/5 % carbon dioxide). The different types of vascular rings were then subjected to a standard normalization procedure and a loading force was applied, mimicking the physiological vessel wall pressure. Following an additional 45-min equilibration period, the myograph chambers were filled with high potassium physiological salt solution (KPSS, 120 mM) to assess vessel viability. After washing, the aortic rings were pre-constricted with increasing concentrations of phenylephrine (Phe, 0.1 nM to 10 μM) to reach approximately 80 % of the maximal KPSS-induced contraction. When a stable contraction-plateau phase was established, vascular tissue was exposed to cumulative concentrations of acetylcholine (ACh, 1 nM to 100 μM) to assess endothelium-dependent relaxation. For endothelium-independent relaxation, cumulative concentration-dependent responses induced by sodium nitroprusside (SNP, 1 nM to 100 μM) were recorded.

### 2.20 Invasive assessment of hemodynamic parameters by Millar catheterization

Invasive assessment of hemodynamic parameters was carried out in mice pre-treated with buprenorphine i.p. (0.1 mg/kg), intubated and anesthetized with isoflurane (3 % induction, 2.5 % maintenance) by using a 1.4 F Millar pressure-conductance catheter (SPR-839, Millar Instrument, Houston, TX, USA) placed into the left ventricle through the right carotid artery as described [57]. The pressure was recorded by a Millar Box and analyzed with Lab Chart 7 (AD Instruments, Oxford, UK).

### 2.21 Induction of AMI and measurement of infarct size and LV function

For inducing myocardial ischemia, mice were treated with buprenorphine (0.05 % i.p), intubated and anesthetized with isoflurane (3 % induction and 2 % maintenance); myocardial ischemia was induced by occlusion of the left anterior descending coronary artery for 45 min followed by 24 h of reperfusion, as previously described [60]. Infarct size was evaluated by staining with 2,3,5-triphenyl tetrazolium chloride (TTC). The area at risk (AAR) and nonischemic areas were evaluated by computer-assisted planimetry. The size of the myocardial infarction is expressed as a percentage of the infarcted tissue area compared to the total AAR. Left ventricular (LV) function was analyzed non-invasively by transthoracic echocardiography before and after AMI in mice anesthetized with 2.5 % isoflurane, as described previously [57]. Left ventricular (LV) end-systolic (ESV) and end-diastolic volumes (EDV), LV ejection fraction (EF), fractional shortening (FS), cardiac output (CO), and stroke volume (SV) were evaluated by using the PSLAX-mode (B-mode) with the manufacturer's software (18–38 MHz; Vevo 2100, Visual Sonics Inc., Toronto, Canada).

### 2.22 Statistical analysis

The sample size was calculated a priori by using G-Power V.3.1 (Heinrich Heine University of Düsseldorf). Statistical analysis was carried out with GraphPad Prism 9 for macOS (Version 9.0.2(134)). Unless stated otherwise, the results are reported as means ± standard deviation (SD). Normal distribution was evaluated by the D'Agostino-Pearson test. Comparisons among multiple groups were performed using 1-way and 2-way analysis of variance (ANOVA) or 2-way repeated measures (RM)-ANOVA, as appropriate, followed by Tukey's or Sidak's post-hoc analysis, as indicated in the figure legends. Where indicated, unpaired Student's t-test with Welch's correction was used to determine if two groups of data were significantly different. The Mann-Whitney test was carried out when data were not normally distributed. p < 0.05 was considered statistically significant. For vessel myography studies, image analysis-based techniques (echocardiography, FMD, Western blot band quantification) the researchers were blinded.

## 3 Results

### 3.1 In human blood, RBCs and WBCs exhibit comparable arginase activity, while mouse RBCs show the lowest arginase activity of all blood cells

To compare arginase activity among blood cell subpopulations in humans and mice, anticoagulated whole blood was separated into plasma, platelets, WBCs, and RBCs (Fig. 1). Platelets were isolated through sequential low-speed centrifugation (150×*g*), while WBCs were separated from RBCs using a leukodepletion filter, achieving >99 % WBC removal as confirmed by flow cytometry (Fig. 1A–C). In humans, WBCs and RBCs exhibited similarly high arginase activity, with values of 4557 ± 2824 and 2357 ± 681 nmoles urea/h/mg protein, respectively, indicating that both cell types contribute substantially to total blood arginase activity (Fig. 1B–Table 1). In RBCs, activity was enriched in membrane fractions (ghosts: 23,370 ± 11,884 nmol urea/h/mg protein) and nearly absent in the cytosol, indicating membrane association (Table 1). In mice, WBCs showed moderate arginase activity (238.7 ± 130.3 nmol urea/h/mg protein) as compared to the much higher levels in human WBCs, while RBC arginase activity was undetectable using the urea assay due to absorbance interference by hemoglobin (Fig. S1). Using an alternative method based on mass spectrometric measurement of ^13^C-l-ornithine formation in RBC ghost preparations we detected a very low arginase activity in RBC ghosts from WT mice (0.013 ± 0.011 nmol l-ornithine/h/mg protein), which was approximately 13,500 fold lower than the activity detected in human RBC ghosts using the same technique (176.2 ± 23.0 nmol l-ornithine/h/mg protein) (Fig. 1D–Table 1). These data show that, while in human blood WBCs and RBCs contribute similarly to total blood arginase activity, in mice WBCs exhibit the highest arginase activity, indicating a species-specific function of arginase in blood cells.

**Table 1:** **Arginase Activity in different blood compartments from mice and humans.** Data are reported as mean ± SD; n = number of human individuals/mice. Welch's *t*-test between WT and RBC Arg1 KO mice, ∗p < 0.05 ∗∗p < 0.01 ∗∗∗p < 0.001 ∗∗∗∗<0.0001; & n = 9; U.D. = under detection limit. We carried out the urea assay in mouse WT RBC lysates, which resulted ca. 35-fold lower than human RBCs; data are included in the table§ but, as can be judged by the SD and by the picture of a representative sample plate in Fig. S1, the reproducibility of the urea assay in mouse RBC lysates is low because of an overwhelming background from hemoglobin (see Fig. S1).

Blood components	Units	Human	WTArg^flox/flox^ mice	RBC Arg KOArg1^flox/flox^ HbbCre ^pos^	p	p
n		5	6	6	**Human vs. WT mice**	**WT mice vs RBC Arg1 KO mice**
Plasma	nmol urea/h/mg protein	6.7 ± 1.3	8.8 ± 2.2^&^	11.6 ± 5.0^&^	0.028∗	0.086
Platelet		109.6 ± 84.6	56.5 ± 45.1	56.9 ± 22.3	0.261	0.986
WBC		4557 ± 2824	238.7 ± 130.3	256.7 ± 153.7	0.027∗	0.823
RBC		2357 ± 681	67.1 ± 39.3^§^	U.D.		
RBC-ghost		23370 ± 11884	U.D.	U.D.		
RBC-ghost	nmol L-ornithine/h/mg protein	176.2 ± 23.0	0.013 ± 0.011	0.0016 ± 0.0028	<0.0001∗∗∗∗	0.0071∗∗

### 3.2 RBC Arg1 KO mice lack Arg1 specifically in erythroid precursors and mature RBCs

**Fig. 2:**
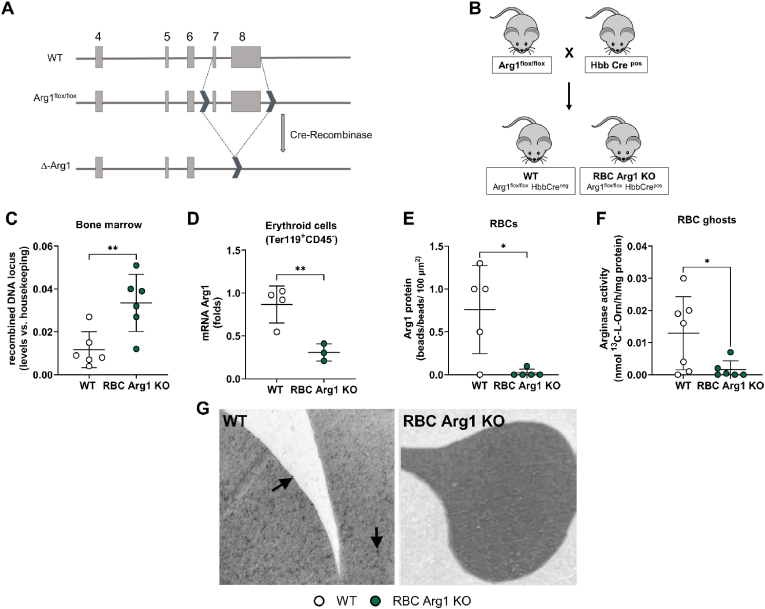
**Generation and characterization of RBC Arg1 KO mice. (A)** Scheme describing the gene-targeting strategy applied to generate Arg1^flox/flox^ mice [48], showing the loxP sequences (black) flanking the exon 7 and 8 of Arg1. Of note, we found that the second loxP sequence is located outside the Arg*1* locus (for full sequence information see Ref. [13]). The Cre-recombinase activity results in excision of exon 7 and 8 and inactivation of Arg1 expression in the targeted cell. **(B)** To generate RBC Arg1 KO mice (Arg1^flox/flox^ Hbb-Cre^pos^) and their respective WT control (Arg1^flox/flox^ Hbb-Cre^neg^), the Arg1^flox/flox^ mice were crossed with Hbb-Cre^pos/neg^ mice to obtain Arg1^flox/flox^ Hbb-Cre^pos/neg^ mice. **(C)** Real-time PCR analysis on DNA isolated from the bone marrow of RBC Arg1 KO/WT mice shows that DNA recombination occurs in the bone marrow of RBC Arg1 KO mice (green), but not of WT controls (white), Welch's *t*-test, ∗∗*p* = 0.0086. **(D)** Real-time reverse transcriptase (RT)-PCR analysis shows loss of mRNA Arg1 expression in the erythroid cells (Ter119^pos^CD45^neg^) isolated from the bone marrow of RBC Arg1 KO mice (green) but not of WT littermate control mice (white), Welch's *t*-test, ∗∗p < 0.01 **(E)** Quantification of immunogold labelling of Arg1 in WT and RBC Arg1 KO mice by transmission electronic microscopy shows low expression of Arg1 in WT RBCs and loss of Arg1 expression in RBC Arg1 KO mice. Welch's *t*-test, ∗p < 0.05. **(F)** Arginase activity determined by mass spectrometry was significantly decreased in RBC ghosts from RBC Arg1 KO as compared to WT littermates. Welch's *t*-test, ∗p < 0.05, ∗∗p < 0.01. **(G)** Electron scanning microscopy with immunogold staining of Arg1 in WT mice (left) and RBC eNOS KO (right).

**Fig. 3:**
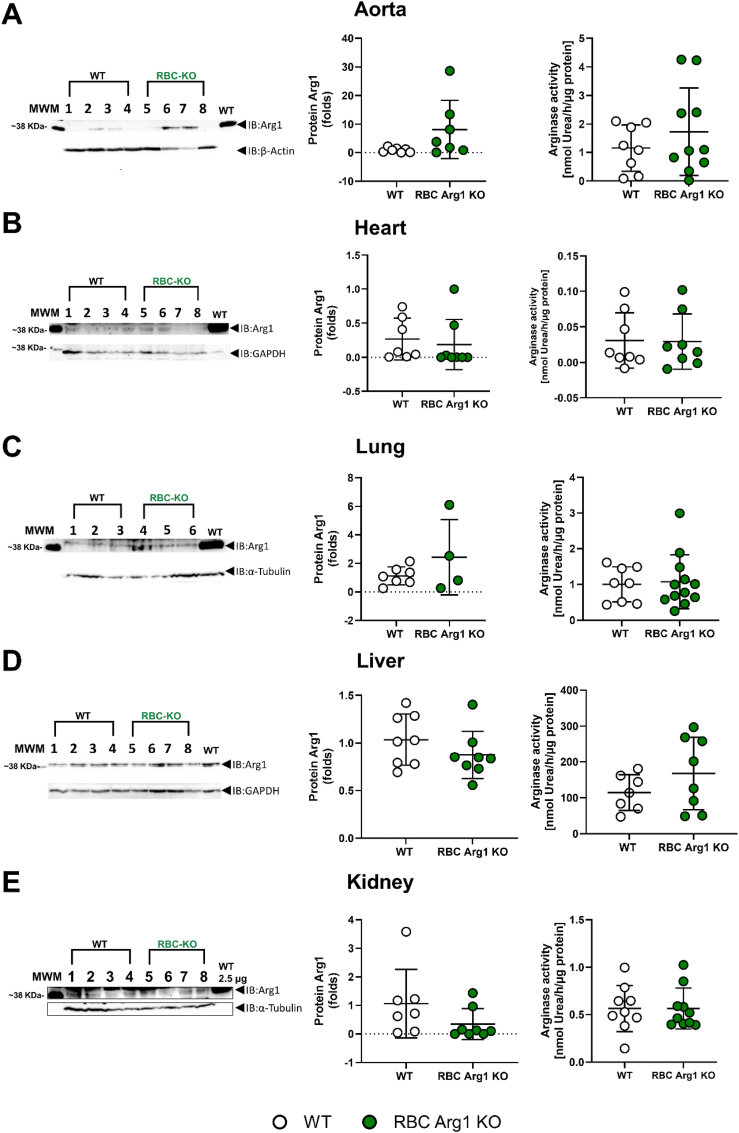
**Expression of Arg1 and arginase activity are preserved in different tissues.** The deletion of Arg1 from erythroid cells does not exert off-target effects in non-targeted tissues including aorta, lung, liver and kidney as determined by Western blotting and colorimetric urea assay. In brackets we indicated the amount of sample that was loaded and for the arginase assay also the incubation time, which reflect the relative abundance of Arg1 in the different tissues. **(A)** Arg1 expression (120 μg) and arginase activity (200 μg protein, 1 h) in aorta lysate. **(B)** Arg1 expression (300 μg) and arginase activity (200 μg protein, 5 h) in heart lysate. **(C)** Arg1 expression (200 μg) and arginase activity (200 μg protein, 40 min) in lung lysate. **(D)** Arg1 expression (5 μg) and arginase activity (2 μg protein, 10 min) in liver lysate. **(E)** Arg1 expression (200 μg) and arginase activity (200 μg, 1 h) in kidney lysate. Comparison between the groups Welch's *t*-test, n.s.

To investigate the pathophysiological role of RBC Arg1 *in vivo* in mice, we generated mice lacking Arg1 specifically in erythroid cells by applying the Cre/loxP system (Fig. 2A), as we did before for eNOS [51]. Homozygous Arg1^flox/flox^ mice [48] - carrying two loxP sequences flanking exon 7 and 8 (for full sequence see Ref. [13]) - were crossed with mice expressing a Cre-recombinase under the control of the Hbb promoter (HbbCre^pos^) [49] (Fig. 2B). DNA recombination of the Arg1 locus was confirmed in the bone marrow of RBC Arg1 KO by testing the presence of the Δ−allele by real-time PCR (Fig. 2C), while WT littermates did not carry the Δ-allele. Erythroid cells (Ter119^+^ CD45^−^) isolated from RBC Arg1 KO mice show a significant decrease of mRNA Arg1 expression as compared to erythroid cells from WT controls (Fig. 2D). The presence/absence of Arg1 in mouse RBC ghosts was difficult to test by Western blotting as the expression of Arg1 in WT was very low and, in many samples, undetectable. The presence/absence of Arg1 in RBCs from WT and RBC Arg1 KO was therefore further examined by transmission electron microscopy. Using this method, we could confirm expression of Arg1 protein in WT mice and its absence in the KOs (Fig. 2E + G). In RBC ghosts arginase activity was detected in WT mice but not in the KOs (Fig. 2F). To test for off-target effects of Cre-recombinase we measured Arg1 expression and activity in aorta, liver, heart, lung, and kidney of RBC Arg1 KO as compared to the same tissues from WT littermate mice, and we found no significant differences between the two groups (Fig. 3).

### 3.3 Lack of Arg1 in erythroid cell precursors does not affect erythroid cell differentiation or blood count

In humans, Arg1 and Arg2 are expressed in erythroid cells [36] and participate to erythroid cell differentiation mainly by regulating polyamine synthesis [61,62]. Therefore, we analyzed whether the lack of Arg1 in erythroid precursor cells may affect terminal erythroid differentiation in the bone marrow or in the spleen in mice. We found no significant changes in terminal erythroid differentiation in the bone marrow of RBC Arg1 KO mice and WT controls as quantified by analyzing the respective percentage of pro-, baso-, poly-, and orthoerythroblasts determined by flow cytometry (Table 2). Moreover, no difference between RBC Arg1 KO and WT mice were observed in the spleen regarding the presence of erythroid precursors and/or spleen size/bodyweight ratio (Table 3). In line with these findings, the percentage of circulating reticulocytes, the concentration of total hemoglobin, and the number of RBCs, WBCs, and platelets did not differ from the WT group (Table 3). The levels of erythropoietin (EPO), or transferrin were unchanged, but we found a significant decrease in plasma ferritin in RBC Arg1 KO mice as compared to WT mice. Thus, in mice the lack of Arg1 in erythroid precursor cells does not affect terminal erythroid cell differentiation although it appears to affect iron storage.

**Table 2:** **Quantitation of terminal erythroid differentiation in the bone marrow and the presence of erythroid precursors in the spleen of RBC Arg1 KO mice and corresponding WT littermate controls**. The proportion of cells at each distinct developmental stage of maturation was normalized based on total nucleated erythroid cells and summarized. All data are expressed as mean ± SD. Welch's *t*-test between WT and RBC Arg1 KO mice, ∗p<0.05

Bone marrow	WT	RBC Arg1 KO	p
	Arg1^flox/flox^ HbbCre^neg^	Arg1^flox/flox^ HbbCre^pos^	
n	5	5	
Proerythroblast (%)	3.77 ± 1.46	4.04 ± 0.89	0.734
Basophilic erythroblast (%)	12.31 ± 1.32	12.47 ± 1.11	0.835
Polychromic erythroblast (%)	24.57 ± 2.00	24.49 ± 1.86	0.954
Orthochromatic erythroblast (%)	59.35 ± 3.11	58.99 ± 2.59	0.846

Spleen	WT	RBC Arg1 KO	p
n	5	5	
Proerythroblast (%)	4.20 ± 2.04	4.76 ± 1.99	0.693
Basophilic erythroblast (%)	12.10 ± 1.00	11.95 ± 1.34	0.855
Polychromic erythroblast (%)	24.24 ± 1.02	23.51 ± 1.82	0.510
Orthochromatic erythroblast (%)	58.20 ± 2.90	59.79 ± 2.14	0.375

**Table 3:** **Blood count of RBC Arg1 KO mice and corresponding WT littermate controls.** Abbreviations: RBC, red blood cells; HGB, hemoglobin; HCT, hematocrit; RDW, RBC distribution width; MCHC, mean corpuscular hemoglobin concentration; MCH, mean corpuscular hemoglobin; MCV, mean corpuscular volume; WBC, white blood cells; Lymph, lymphocytes; Mo, monocytes; Neu, Neutrophils; PLT, platelet count; MPV, mean platelet volume. All data are expressed as mean ± SD. Welch's *t*-test between WT and RBC Arg1 KO mice.

Parameter	Units	WT	RBC Arg1 KO	p
		Arg1^flox/flox^ HbbCre^neg^	Arg1^flox/flox^ HbbCre^pos^	
n		8	9	
Red blood cell count				
RBC	10^12^/L	9.40.6	8.9 ± 0.8	0.128
HGB	g/dl	13.0 ± 0.5	12.5 ± 1.6	0.443
HCT	%	40.0 ± 2.4	37.2 ± 4.0	0.101
Red blood cell indexes				
RDW	%	20.0 ± 1.1	19.5 ± 0.7	0.376
MCHC	g/dl	32.6 ± 2.4	33.7 ± 1.1	0.290
MCH	pg	13.8 ± 1.1	13.9 ± 0.8	0.857
MCV	fl	42.3 ± 0.7	42.0 ± 0.9	0.523
White blood cell count				
WBC	10^9^/L	3.0 ± 1.3	3.5 ± 1.4	0.423
Lymph	10^9^/L	2.0 ± 0.8	2.1 ± 0.8	0.868
Lymph	%	70.9 ± 15.7	61.5 ± 22.8	0.335
Mo	10^9^/L	0.2 ± 0.1	0.2 ± 0.1	0.234
Mo	%	4.8 ± 1.8	6.0 ± 2.8	0.314
Neu	10^9^/L	0.8 ± 0.6	1.2 ± 0.9	0.302
Neu	%	24.2 ± 14.6	32.5 ± 20.1	0.344
Platelet count				
PLT	10^9^/L	499.3 ± 58.1	446.4 ± 62.1	0.090
MPV	fl	6.0 ± 0.4	6.1 ± 0.3	0.413
Plasma				
Erythropoietin	pg/mL	39.7 ± 13.5	43.2 ± 4.50	0.548
Ferritin	mg/mL	814 ± 326	449 ± 285	0.037∗
Transferrin	mg/mL	4.27 ± 0.9	3.34 ± 0.6	0.055
Spleen				
Spleen/BW ratio	mg/g	3.1 ± 0.4	3.2 ± 0.4	0.594

### 3.4 RBC Arg1 KO mice display preserved systemic arginine bioavailability and NO metabolites

We further investigated whether the lack of Arg1 in mature circulating RBCs affects l-arginine bioavailability and NO metabolite levels in blood of RBC Arg1 KO mice as compared to WT mice by measuring the levels of l-arginine, l-ornithine, and l-citrulline in the plasma of RBCs Arg1 KO mice and WT controls by LC-MS (Table 4). We found that l-ornithine levels were decreased in plasma of RBC Arg1 KO mice as compared to WT mice; instead, the levels of l-arginine, l-citrulline, as well as the bioavailability of l-arginine (calculated as the ratio of l-arginine/(l-ornithine + l-citrulline)) [63]) did not show any significant difference between RBC Arg1 KO and WT mice. This is consistent with the observation that primates with Arg1 mutation in RBCs only but preserved Arg1 in liver did not show changes in plasma l-arginine levels [38]. The levels of l-arginine metabolites found in the WT controls are in line with those reported by other studies [64]. We also determined the concentrations of NO metabolites in blood and tissues/organs by gas-phase chemiluminescence detection (Table 5). With one exception, the concentration of nitrite in aortic vascular tissue, the levels of NO metabolites were preserved in RBC Arg1 KO as compared to WT littermates for all compartments analyzed. These data show that lack of Arg1 specifically in RBCs affects l-ornithine levels in plasma but neither affects systemic arginine bioavailability nor circulating NO metabolites. What accounts for the increased tissue concentration of nitrite in RBC Arg1 KO compared to WT remains unclear.

**Table 4:** **Determination of****l****-arginine metabolites in plasma of RBC Arg1 KO mice and WT littermates.** The l-arginine bioavailability was calculated as the ratio of L-Arg/(L-Orn + L-Cit). All data are expressed as means ± SD. Welch's *t*-test between WT and RBC Arg1 KO mice, ∗p˂0.05.

	WT	RBC Arg1 KO	p
	Arg1^flox/flox^ HbbCre^neg^	Arg1^flox/flox^ HbbCre^pos^	
n	8	12	
l-arginine	137.0 ± 22.46	122.1 ± 24.44	0.179
l-ornithine	68.47 ± 7.84	59.57 ± 8.55	0.029∗
l-citrulline	31.52 ± 1.38	31.36 ± 1.22	0.789
l-arginine bioavailability	1.37 ± 0.17	1.34 ± 0.17	0.696

**Table 5:** **NO metabolites in blood and organs of RBC Arg1 KO and corresponding WT littermate controls**. All data are expressed as mean ± SD. Welch's *t*-test between WT and RBC Arg1 KO mice ∗ p < 0.05

		WT	RBC Arg1 KO	
Metabolite		Arg1^flox/flox^ HbbCre^neg^	Arg1^flox/flox^ HbbCre^pos^	p
n		8	8	
Heart				
Nitrite	μM	1.56 ± 1.68	3.70 ± 4.61	0.2521
RXNO	nM	32.86 ± 15.43	36.00 ± 21.26	0.7470
NO-heme	nM	19.03 ± 12.25	31.86 ± 26.99	0.2542
Total NO species	μM	1.61 ± 1.68	3.77 ± 4.62	0.2496
Lung				
Nitrite	μM	1.96 ± 0.82	2.08 ± 0.69	0.7763
RXNO	nM	30.15 ± 13.96	32.46 ± 21.43	0.8030
NO-heme	nM	10.74 ± 4.38	8.06 ± 6.00	0.3267
Total NO species	μM	2.00 ± 0.81	2.12 ± 0.70	0.7704
Liver				
Nitrite	μM	1.44 ± 1.24	2.33 ± 2.42	0.3929
RXNO	nM	559.53 ± 157.60	521.45 ± 132.99	0.6341
NO-heme	nM	284.48 ± 238.25	261.30 ± 219.82	0.8426
Total NO species	μM			
Aorta				
Nitrite	μM	1.59 ± 1.49	4.79 ± 2.58	0.0183∗
RXNO	nM	71.01 ± 33.02	66.28 ± 30.12	0.7691
HbNO	nM	30.04 ± 9.49	21.90 ± 11.11	0.1377
Total NO species	μM	1.70 ± 1.50	4.88 ± 2.58	0.0189∗
Plasma				
Nitrite	μM	0.55 ± 0.33	0.47 ± 0.23	0.6301
RXNO	nM	14.56 ± 10.60	31.66 ± 27.28	0.1946
Total NO species	μM	0.56 ± 0.33	0.49 ± 0.23	0.6467
Erythrocytes				
Nitrite	μM	0.51 ± 0.15	0.36 ± 0.23	0.1725
RXNO	nM	317.19 ± 248.60	282.23 ± 220.87	0.7706
NO-heme	nM	2.18 ± 0.90	2.02 ± 1.49	0.7923
Total NO species	μM	0.82 ± 0.27	0.65 ± 0.45	0.3778

### 3.5 Preserved vascular function and systemic hemodynamics

**Fig. 4:**
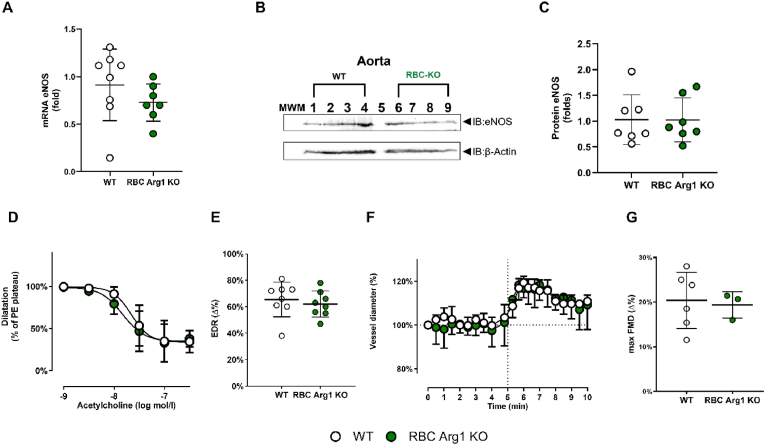
**Vascular endothelial dilator function is fully preserved in RBC Arg1 KO mice (A**–**C)** Preserved mRNA (**A**) and protein (B, C) of eNOS (ca 135 kDa) in the aorta of RBC Arg1 KO vs. WT littermates determined by qPCR and Western blot; (**B)** representative Western blot; (**C**) band intensity quantification vs. β-actin (Welch's T-test, n.s.). **(D**–**E)** Nitric oxide–dependent vascular endothelial function is fully preserved in RBC Arg1 KO mice compared with the respective WT littermate controls. **(D)** Preconstricted aortic rings from RBC Arg1 KO show a fully preserved acetylcholine (ACh)-induced vasodilation (n = 8 per group; 2-way repeated measurement [RM]-ANOVA genotype p = 0.4834, concentration *P* < 0.0001; post hoc Šídák's multiple comparisons test) **(E)** Endothelium-dependent relaxation (EDR) in response to ACh (calculated as the percentage of the maximal ACh response) is fully preserved in RBC Arg1 KO compared with their respective WT controls (Welch's T-test n.s.). **(F**–**G)** Flow-mediated dilation (FMD) of the iliac artery is fully preserved in RBC Arg1 KO (n = 3) mice vs. WT (n = 6) littermate, (mixed-effects model (REML) genotype p = 0.8390, time p < 0.0001; post hoc Tukey) **(F)** The changes in vessel diameters were assessed *in vivo* by ultrasound (Welch's *t*-test). **(G)** Maximal FMD was calculated as the percentage of maximal flow-mediated dilatatory response measured in RBC Arg1 KO mice (n = 3) and WT littermate controls (n = 6) (Welch's *t*-test, n.s.).

To verify if lack of RBC Arg1 may affect vascular function and systemic hemodynamics, we investigated eNOS expression in the aorta and vascular reactivity of aortic rings *ex vivo* and endothelial function by FMD of the iliac artery *in vivo* (Fig. 4). We found that eNOS expression (mRNA + protein) in the aorta was preserved in RBC Arg1 KO as compared to the WT (Fig. 4A, B, C). Accordingly, endothelium-dependent relaxation in response to ACh or contractile response to Phe were not different in aortic rings from RBC Arg1 KO mice as compared to vessels from WT littermates (Fig. 4D–E and Fig. S2). Similarly, FMD responses were not different in RBC Arg1 KO mice and WT littermate controls (Fig. 4F–G). In addition, cardiovascular systemic hemodynamics was determined invasively by Millar catheter. Heart rate (HR), systolic, diastolic and mean arterial pressure were preserved in RBC Arg1 KO mice as compared to WT littermates (Table 6). Taken together, RBC Arg1 KO revealed a fully preserved vascular endothelial function and cardiovascular hemodynamics.

**Table 6:** **Systemic hemodynamics as assessed by the Millar catheter**. DBP, diastolic blood pressure; SBP systolic blood pressure; MAP, mean arterial pressure; HR, heart rat; All data are expressed as mean ± SD. Welch's *t*-test between WT and RBC Arg1 KO mice.

	WT		RBC Arg1 KO		
	Arg1^flox/flox^ HbbCre^neg^		Arg1^flox/flox^ HbbCre^pos^		
	Mean ± SD	n	Mean ± SD	n	p
**DBP**	64 ± 11	20	64 ± 7	19	0.906
**SBP**	91 ± 11	20	96 ± 11	19	0.129
**MAP**	72 ± 11	20	75 ± 8	19	0.140
**HR**	503 ± 64	20	472 ± 64	19	0.602

### 3.6 Infarct size and LV dysfunction after AMI are unchanged in RBC Arg1 KO mice

**Fig. 5:**
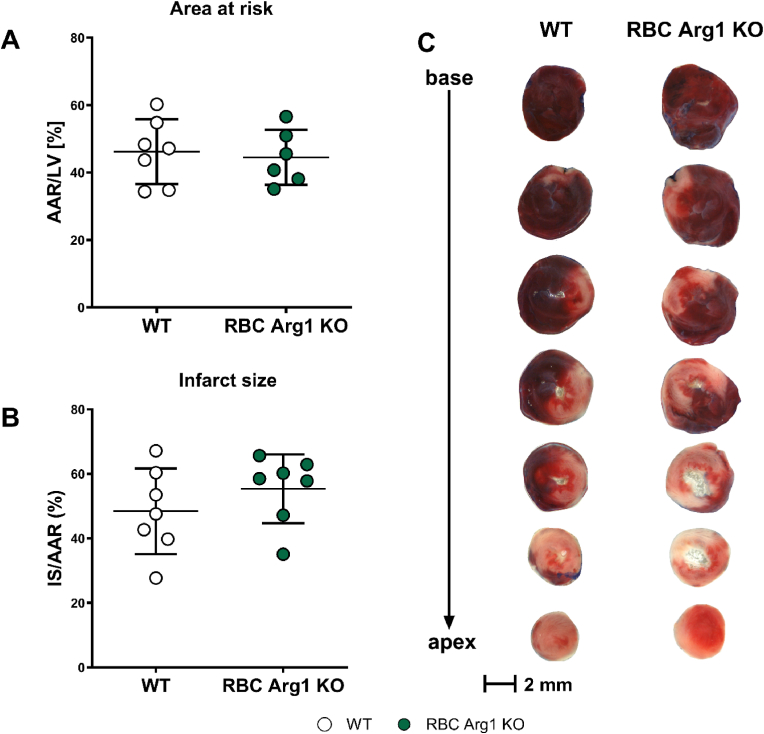
**Infarct size after acute myocardial infarction is not affected by inactivation of Arg1 in RBCs.** The percentage of **(A)** area at risk (AAR) and **(B)** infarct size/area at risk (IS/AAR) were analyzed in RBC Arg1 KO (n = 7) and WT littermate controls (n = 6) after 45 min of ischemia induced by ligation of the left anterior descending (LAD) coronary artery and 24 h reperfusion (Welch's *t*-test, n.s) **(C)** Exemplary image of heart cross-sections with TTC staining.

To test whether RBC Arg1 KO may affect the outcome of AMI *in vivo* in mice, RBC Arg1 KO mice and their WT littermates underwent 45 min open-chest coronary occlusion followed by 24 h of reperfusion (Fig. 5). AAR did not differ between the groups (Fig. 5A). In RBC Arg1 KO mice, infarct size was not significantly different from that in respective littermate WT controls (Fig. 5B green vs. white)**.** The effects of AMI and RBC-Arg1 KO on LV function *in vivo* were determined by echocardiography measurements carried out in all mice before and 24 h after induction of AMI (Table 7). We did not find any significant differences in CO, SV, HR, EF or FS at baseline and after AMI. These data provide compelling evidence that Arg1 expressed in RBCs *per se* does not modulate infarct size or LV function after AMI *in vivo* in mice.

**Table 7:** **Echocardiographic parameters were assessed in RBC Arg1 KO mice by high-resolution ultrasound before and after AMI.** Data are reported as mean ± SD; n = number of mice. Differences between KO/WT mice pre- and post-AMI were calculated by Welch's *t*-test. ∗p < 0.05 (shown in the table). HR, heart rate; CO, cardiac output; SV, stroke volume; EF, ejection fraction; FS, fractional shortening; ESV, end-systolic volume; EDV, end-diastolic volume.

Parameter	Baseline				Post-AMI		
	Unit	WT	RBC Arg1 KO	p	WT	RBC Arg1 KO	p
		Arg1^flox/flox^	Arg1^flox/flox^ HbbCre^pos^		Arg1^flox/flox^	Arg1^flox/flox^ HbbCre^pos^	
	n	7	7		7	7	
HR	bpm	467 ± 50	458 ± 38	0.718	500 ± 37	478 ± 77	0.510
CO	ml/min	18 ± 6	17 ± 2	0.656	13 ± 3	15 ± 5	0.329
SV	μL	38 ± 10	37 ± 4	0.817	25 ± 6	31 ± 9	0.209
EF	%	44 ± 8	46 ± 5	0.448	38 ± 6	47 ± 10	0.051
FS	%	11 ± 3	14 ± 3	0.088	8 ± 4	14 ± 7	0.410
ESV	μL	47 ± 6	44 ± 9	0.432	42 ± 10	35 ± 11	0.230
EDV	μL	84 ± 11	80 ± 10	0.470	68 ± 14	66 ± 14	0.827

## 4 Discussion

This study was undertaken to understand the role of RBC Arg1 *in vivo* with regard to control of systemic arginine and NO bioavailability, systemic hemodynamics and cardioprotection. The major findings of this study are: (1) While in humans both RBCs and WBCs contribute significantly to total blood arginase activity, with WBCs showing approximately twofold higher activity than RBCs (WBC > RBC ≫ platelets (PLTs) > plasma), in mice WBCs are the dominant source of arginase activity, with negligible contribution from RBCs (WBC ≫ PLTs > plasma). (2) Arginase activity in human RBCs is primarily localized to the membrane (ghost) fraction rather than the cytoplasmic compartment as in hepatocytes. (3) Arginase activity (as assessed by l-ornithine formation) in human RBC membranes is over 13,500-fold higher than in mouse RBC membranes, where it is expressed at very low levels. (4) RBC Arg1 KO mice exhibit preserved systemic l-arginine and NO bioavailability, vascular eNOS expression, conduit vessel function, and blood pressure. (5) Following AMI, RBC Arg1 KO mice show preserved LV function and unchanged infarct size compared to littermate WT controls. Taken together, these findings demonstrate a species-specific distribution and function of Arg1 in blood cells. In contrast to observations in human cohorts, in mice RBC Arg1 does not regulate systemic L-arginine–NO bioavailability or confer cardioprotection.

Arg1 is commonly known as a soluble liver enzyme mainly found in the cytoplasm of hepatocytes, where it was identified and isolated for the first time [65]. Therefore, we were expecting to find Arg1 in the soluble cytoplasmatic fraction of human RBCs. Instead, we were surprised to see that in human RBCs arginase activity was mainly located in the membrane fraction (RBC ghosts) of leukodepleted human RBCs rather than in the cytoplasmic fraction. Thus, we found that human RBC ghost preparations show an activity of 23,370 ± 11,884 nmol urea/h/mg protein, while RBC lysates show an activity of 2,357 ± 681nmol urea/h/mg protein, with no detectable urea formation in the cytoplasmatic fraction (i.e. the supernatant of RBC ghosts). Others obtained similar results in human samples [66]. Interestingly, by using recombinant His-6-arginase 1 and His-6-flotillin-1, these authors found that Arg1 can interact with flotillin-1, which leads to and increased arginase activity [66]. Since flotillin-1 is found in the lipid rafts of human erythrocytes [67], they speculated that flotillin-1 may participate in Arg1 trafficking and may contribute to regulating its function in human RBCs.

Human and other primate show very high arginase activity in RBCs [35]; in human WBCs constitutive arginase activity has only been described to be present in the granula of human polymorphonuclear (PMN) cells and not in other WBC subpopulations [68]. Therefore, in human blood we were expecting the highest arginase activity in the RBC fraction rather than other blood cell subpopulations. Instead, the arginase activity (determined as urea formation) in the WBC fraction and RBC fraction are comparable (in the WBC fraction it was 2-fold higher than in RBCs, but the number of subjects we investigated herewas low). Human RBCs and WBCs show a 20-fold–40-fold higher arginase activity than in platelets or in plasma, where it was barely detectable.

Like in human blood, also in mouse blood the highest arginase activity among blood cell subpopulations was localized into the WBC fraction (WBC ≫ PLTs > plasma). However, this similarity is only apparent. As mentionedabove, PMN constitutively express Arg1 in their granula, and in these cells the expression of Arg1 is not modulated by Th2 cytokines [68]. Insteadin mouse blood, Arg1 is predominantly expressed by myeloid-derived suppressor cells (granulocytic and monocytic), with inducible expression in monocytes, macrophages, and neutrophils, which occurs particularly in inflammatory or disease conditions; lymphoid cells do not express Arg1 (evidence summarized in Table 2 of reference [69]). Nevertheless, in both species human and mouse Arg1 was shown to play an immunomodulatory role via modulation of T-cell activation [1,17,69,70].

In contrast to human blood, in mouse RBCs expression and activity of Arg1 were very low and their measurement was particolarly challenging because of the presence of overwhelming concentrations of hemoglobin. Nevertheless, as quantified by immunogold labelling and TEM, the presence of Arg1 was detected in RBCs in WT mice at very low abundance and was around 6-fold less as compared to mouse ECs (Fig. S3). By analyzing the conversion of ^13^C-l-arginine into ^13^C-l-ornithine by human and mouse RBC ghosts by using mass spectrometry, we found 13,500-fold lower activity in mouse RBC ghosts as compared to human RBC ghosts; in RBC ghosts from RBC Arg1 KO mice the activity was at the limit of detection or below. Altogether, these results clearly shows that Arg1 is present and active in mouse RBCs, albeit at very low levels, and that arginase activity in blood is mainly found in WBCs and not in RBCs.

In our hands, the classical urea assay was not suitable to detect arginase activity in mouse RBC lysates or RBC ghosts. To analyze arginase activity in mouse RBC lysate one needed to load ca. 1–3 mg of protein, of which 99 % consist of hemoglobin; the methemoglobin spectrum overlap with the absorbance of the urea adduct with α-isonitrosopropiophenone with A at *λ*_max_ = 540 nm, making it challenging to reliably determine urea concentrations photometrically (please see a representative picture of the plate and the spectra in the Supplement Fig. S1). Arginase activity in ghosts from WT mice (prepared after leukodepletion of the blood by filtration) was around or below the limit of detection of the assay (5 nmoles urea/h/mg protein as compared to a heat-inactivated sample) and experiments were not satisfactorily reproducible (data were therefore omitted from Fig. 1B).

These observations are comparable to earlier studies carried out by using radioactive tracing of l-arginine to l-ornithine conversion [35,39,45]. In these studies, arginase activity could be detected at high levels in hemolysates from humans and primates (957 ± 206 μmol urea/g hemoglobin/h), but the arginase activity in hemolysates from rodents (mouse and rats) was below 1 μmol urea/g hemoglobin/h [35].

The findings of a comparable WBC and RBC arginase activity in human blood, and much higher arginase activity in rodent WBCs vs. RBCs are also important from a technical point of view. Indeed, WBCs are common contaminants of RBC preparations, as emphasized elsewhere [71]; therefore, leukodepletion needs to be carried out when measuring arginase activity in RBC suspensions. We became aware of this issue when we were carrying out experiments to verify the lack of eNOS expression in RBCs from RBC eNOS KO vs. WT mice by immunoprecipitation; without leukodepletion we observed an eNOS band in the RBC eNOS KO sample, which was absent after leukodepletion [51].

From an evolutionary point of view, arginases are more ancient enzymes than NOS isoforms, and rodents are evolutionarily older than primates. This raises the question: why do primates express high levels of arginase in RBCs, whereas rodents, cats, and dogs show very low expression? Interestingly, more evolutionarily recent enzymes are often well conserved across species. For example, in our own studies, we observed that eNOS and sGC—both of which are present in mouse and human RBCs—are conserved across species [51,72,73]. Already in 1985, the group of Cederbaum proposed that the expression of arginase in RBCs of humans and other primates resulted from a regulatory alteration that evolved under positive selection pressure, rather than being a “vestigial presence of an arcane function” [35]. The nature of this selective pressure remains unclear, i.e., whether arginase expression in RBCs confers any distinct advantage or disadvantage to the host organism. One possible function may be immunomodulation. Recently, it was shown that human erythroid precursor cells (CD71^high^ CD235^mid^) express both Arg1 and Arg2 and are potent inhibitors of T-cell proliferation; however, they lose these properties at later stages of differentiation and in disease [62].

We also investigated the hypothesis that Arg1 in erythroid cells contributes to the regulation of erythropoiesis. In human bone marrow, Arg1 is upregulated at very late stages of terminal differentiation [61,62], and l-arginine is primarily required for polyamine biosynthesis and hypusination of the eIF5 transcription factor, rather than for enzymatic synthesis of creatine or NO [74,75]. Mouse pro-erythroblasts express lower levels of Arg1 compared to humans [62,76]. Based on the data reported here, we conclude that the absence of Arg1 in erythroid precursor cells does not affect erythroid differentiation in the bone marrow or spleen (which contribute approximately 80 % and 20 % of erythropoiesis in adult mice, respectively), nor does it alter peripheral blood counts. Thus, Arg1 appears to play only a minor role in erythroid differentiation in the mouse and/or its absence may be fully compensated by mitochondrial Arg2 activity in precursor cells. However, we can exclude a compensatory role of Arg2 in mature RBCs, as arginase activity assays, while not isoform-specific, show no detectable activity in these cells.

A mild decrease in ferritin, and potentially transferrin, in the absence of erythroid Arg1 in RBC Arg1 KO mice suggests a role for Arg1 in iron handling, warranting further investigation of the potential role of Arg1 under conditions such as iron-deficiency anemia, altered erythropoiesis, infection, or chronic inflammatory states, where the effects may be more pronounced.

The very low expression and activity of Arg1 in mouse RBCs also explain the fully preserved l-arginine/NO levels, vascular function and systemic hemodynamics in RBC Arg1 KO mice as compared to WT mice. Human and non-human primates with total body Arg1 mutations or mice with global or liver specific Arg1 KO show hyperarginemia [[23], [24], [25], [26], [27], [28], [29], [30], [31], [32], [33]], indicating that the lack of liver Arg1 (rather than RBC Arg1) induces hyperarginemia. Interestingly, a preserved l-arginine bioavailability in plasma in RBC Arg1 KO mice is consistent with the observation that primates with Arg1 mutation in RBCs only (but preserved Arg1 in liver) did not show changes in plasma L-arginine levels [38]; the authors proposed that when arginase activity is incapsulated in RBCs it does not affect L-arginine plasma levels. Accordingly, in patients with sickle cell disease the increased arginase activity in plasma, which occurs as a consequence of a hemolytic crisis, causes both NO scavenging by free hemoglobin and l-arginine depletion by free arginase [44,45,77]. Thus, the RBC membrane appears to compartmentalize arginase preserving circulating l-arginine levels.

Consistent with these findings, we found that RBC Arg1 KO mice also showed a fully preserved vascular eNOS expression and conduit vessel function *ex vivo* and *in vivo*. Under homeostatic conditions there were no changes in HR, CO and systolic/diastolic blood pressure, indicating that peripheral resistance and LV-function were also preserved at baseline under homeostatic conditions. After AMI, RBC Arg1 KO mice show preserved LV function and no changes in infarct size as compared to the respective littermate controls. The lack of cardioprotection after AMI in RBC Arg1 KO mice as compared to WT was somehow unexpected. Previously, it was shown that arginase inhibition potentiates cardioprotective effects of human, rat and mouse RBCs on ischemia/reperfusion injury as determined in Langedorff heart *ex vivo* bioassay [41]. These cardioprotective effects of human RBCs are also decreased in patients with diabetes type 2, where arginase activity in RBCs is increased [78]. As discussed, Arg1 levels in WT RBCs are extremely low under physiological conditions. Therefore, the absence of a clear phenotype in RBC Arg1 KO mice may thus reflect this low baseline expression under homeostatic healthy conditions and the lack of disease conditions like diabetes, cancer or infection. It remains to be explored whether erythroid Arg1 overexpression in mice would instead confer immunomodulatory protective effects or instead exacerbate infection or cancer outcomes by inhibiting T-cell responses.

Interestingly, in another study, RBC Arg1 KO mice (generated by using a erythropoietin receptor (Epo-R) promoter-dependent Cre-recombinase), crossed with ApoE KO and fed a high fat atherogenic diet showed increased vessel calcification as compared to the Cre negative littermate mice [47]. This phenotype is likely related to upregulation of Arg1 in pro-inflammatory/atherogenic conditions, which unmasks the role of Arg1 (similar to other endothelial specific models). Importantly, the authors excluded off-targets effects of the Epo-R promoter (which is expressed also in tissue macrophages and hematopoietic cells [79]) by analyzing DNA recombination in the targeted cell by crossing the mice with mT/mG mice [47,80]. In this study, we used the HbbCre positive mice to generate RBC-specific Arg1 KO mice. The HbbCre positive mice carry a construct obtained by combining of the promoter region controlling the expression of the β-globin gene with the β-globin locus control region, which restricts the expression of the Cre-recombinase in erythroid cells [49].

Taken together, the result of the present study demonstrates that activity, distribution and function of arginase in the blood differ profoundly between humans and mice. In humans, the activity of arginase plays an important role in erythroid precursor cell differentiation and/or mature erythrocytes function, and is modified in hemoglobinopathies and hematological diseases [1]. In striking contrast, in mice, the activity of arginase does not influence RBC differentiation and does not affect systemic l-arginine bioavailability and cardioprotection after AMI. Given the fundamental importance of arginases in human disease and the need for more effective pharmacological interventions [1], these findings underscore the importance and necessity of using human specimens and of conducting clinical studies, rather than relying on experimental mouse models of arginase deficiency. Only in human specimens, 3D cultures, organoids and studies will be possible to address unresolved questions about the role of Arg1 im erythroid cells and to develop more effective arginase-targeted therapies in humans.

## CRediT authorship contribution statement

**Sophia K. Heuser:** Writing – review & editing, Writing – original draft, Visualization, Investigation, Formal analysis. **Junjie Li:** Writing – review & editing, Writing – original draft, Visualization, Methodology, Investigation, Formal analysis. **Zhixin Li:** Writing – review & editing, Investigation, Formal analysis. **Anthea LoBue:** Writing – review & editing, Investigation, Data curation. **Kyle Heard:** Writing – review & editing, Investigation, Data curation. **Julia Hocks:** Investigation, Formal analysis. **Tatsiana Suvorava:** Writing – review & editing, Project administration, Investigation. **Ron-Patrick Cadeddu:** Investigation. **Corinna Strupp:** Investigation. **Luke Dunaway:** Writing – review & editing, Investigation. **Zhengbing Zhuge:** Investigation. **Stacy L. Gelhaus:** Writing – review & editing, Investigation. **André Heinen:** Writing – review & editing. **Ulrich Germing:** Writing – review & editing. **Martin Feelisch:** Writing – review & editing, Investigation. **Mattias Carlström:** Writing – review & editing. **Brant Isakson:** Writing – review & editing, Data curation. **Malte Kelm:** Writing – review & editing. **Jon O. Lundberg:** Writing – review & editing. **Miriam M. Cortese-Krott:** Writing – review & editing, Writing – original draft, Supervision, Resources, Project administration, Methodology, Investigation, Funding acquisition, Formal analysis, Data curation, Conceptualization.

## Declaration of competing interest

The authors declare that they have no known competing financial interests or personal relationships that could have appeared to influence the work reported in this paper.

## Data Availability

Data will be made available on request.
