# Supplementary material for: Divergent roles of red cell arginase in humans and mice: RBC Arg1 KO mice show preserved systemic l-arginine bioavailability and infarct size in vivo
Source: Redox Biol. 2025 Jul 14;86:103768. doi: 10.1016/j.redox.2025.103768 (PMC12328693; doi:10.1016/j.redox.2025.103768)
Supplement: Multimedia component 1 [file mmc1.docx]

**Supplementary data**

**Supplementary Figures**

**Figure S1**


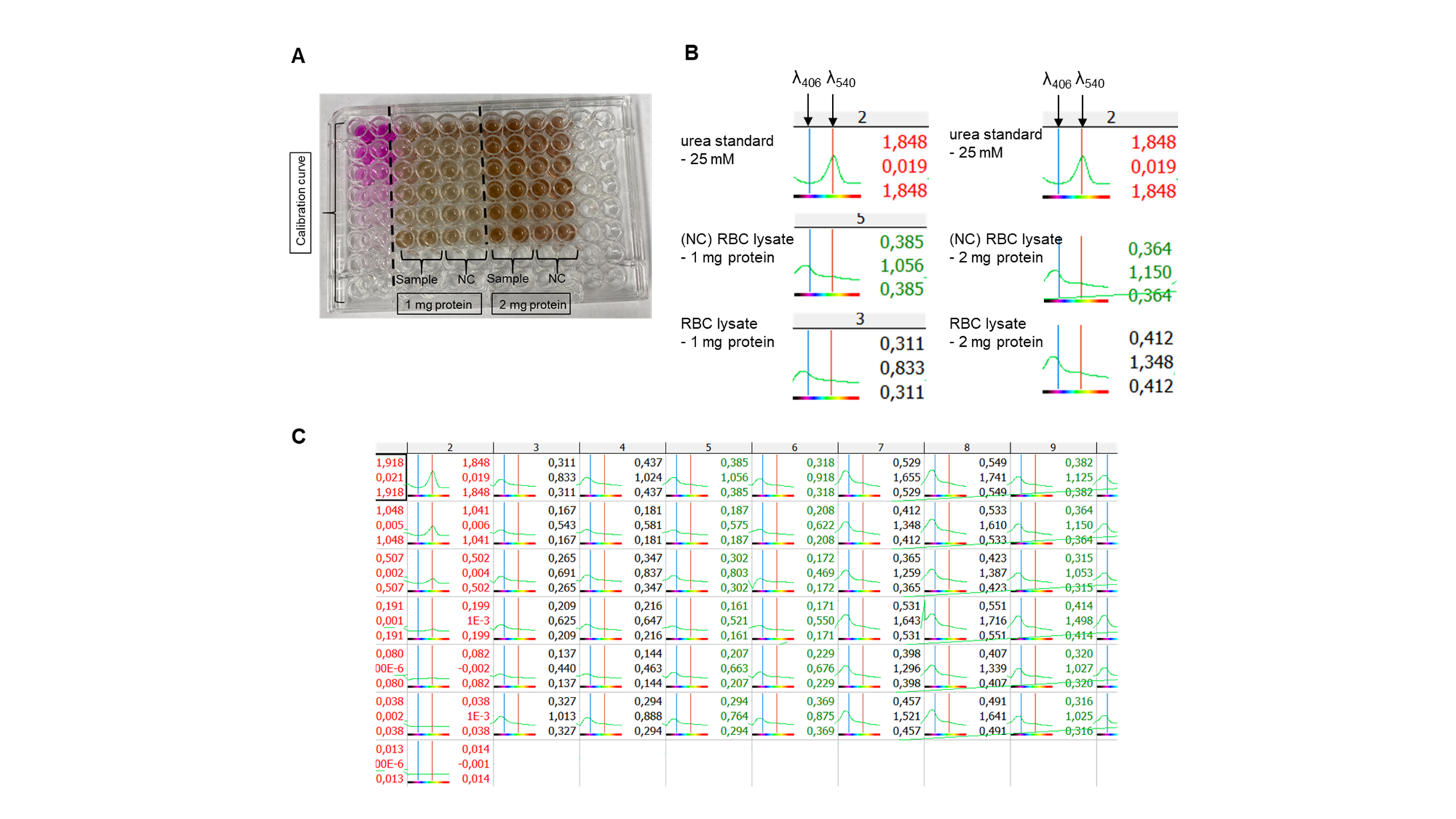


**Figure S1. Detection of the urea adduct with α-isonitrosopropiophenone with absobtion at λmax = 540 nm in mouse RBC lysates by UV-vis.** The calibration curve is prepared in buffer by reacting a range of urea standards (0.4 to 25 mM) with α-isonitrosopropiophenone in acid at 100°C. RBC lysates (1 mg and 2 mg total proteins) and respective heat inactivated background controls (negative control, NC) are reacted with 500 mM L-arginine at 37°C in the presence of Mn^2+^ for 1 hour and the urea formed is reacted with 508 mM α-isonitrosopropiophenone in acid at 100°C. Under these conditions we observe protein oxidation (from red oxyhemoglobin to brown methemoglobin) and precipitation (presence of flocculates in the suspension); samples are then cleared by centrifugation at max speed for 1 min and supernatants are loaded on a 96 well plate. **(A)** Exemplary picture showing the standard curve in buffer, the samples and their respective heat inactivated controls (NC) with total protein amounts of 1 mg and 2 mg. **(B+C)** UV-vis spectra (from 315 nm to 700 nM) of urea standards (from 25 mM to 0.4 mM in duplicates), urea from RBC lysates and their respective inactivated controls (NC) with total protein amount of 1 mg and 2 mg; the adducts of urea with α-isonitrosopropiophenone in RBC lysates cannot be quantified due to overlapping of the α-isonitrosopropiophenone absorbance peaks (λ_max_ ≈ 540 nm) with the methemoglobin Soret band (λ_max_ ≈ 406 nm).

**Figure S2**


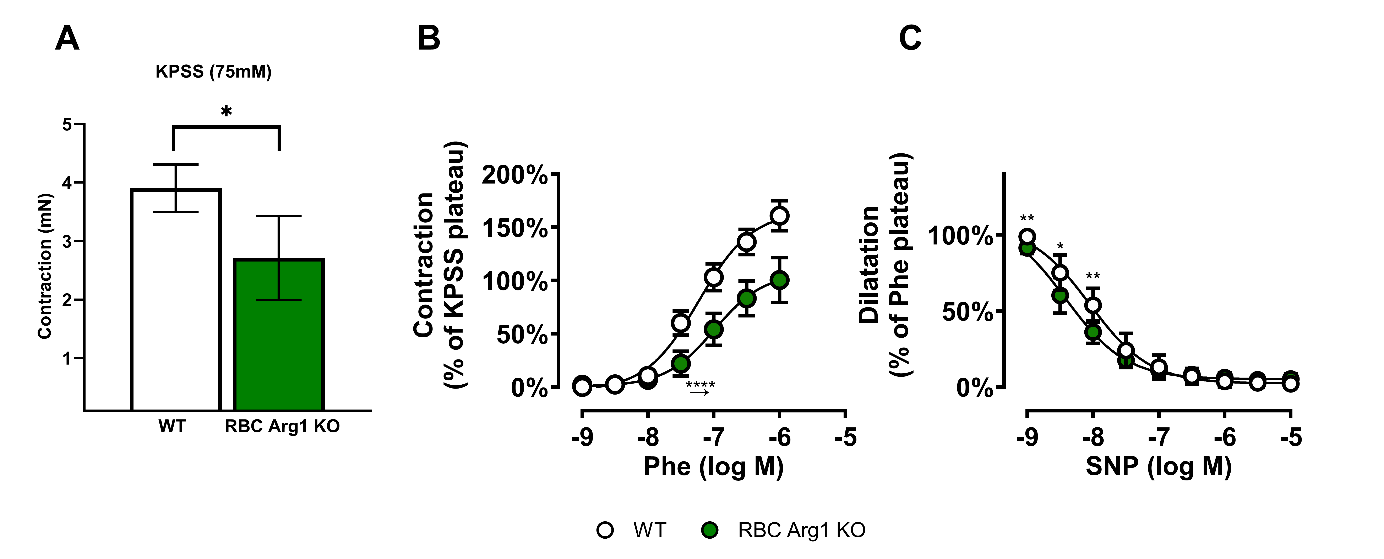


**Figure S21 Vascular endothelial function of aortic rings from RBC Arg1 KO (SNP and PE responses)**. **(A)** RBC Arg1 KO mice showed a significant decrease in the contractility towards KPSS (n=8 per group Welch’s, t-test, **p < 0.01) **(B)** aortic rings of RBC Arg1 KO mice show a significant decrease in contractility response to increasing phenylephrine (Phe) concentrations compared to WT littermate controls. (n=8 per group; 2-way repeated measurement [RM]-ANOVA p<0.0001; Tukey ****p<0,001) **(C)** The vasodilatory response of aortic rings to increasing concentrations of the NO donor sodium nitroprusside (SNP) is increased in RBC Arg1 KO mice as compared to their littermate WT controls. 2-way ANOVA p<0.0001; Sidak’s *p<0.05, **p<0.01, ***p<0.005.

**Figure S3**

**Figure S3 Quantification of immunotransmission electronic microscopy with immunogold labelling of Arg1 in the endothelium of WT mice and of RBC of WT RBC Arg1 KO mice.** RBCs of WT showed 6-fold less levels of Arg1 compared to the endothelium of WT mice. Ordinary one-way ANOVA p<0.001, Tukey **** p<0.0001; Welch’s t-test # p<0,05

**Supplementary Tables**

**Table S1. Specific primer for DNA recombination of RBC Arg1 KO mice designed for each gene (Transnetyx, Cordova, TN, USA)**

| Gene | Forward Primer | Reverse Primer |
| --- | --- | --- |
| ∆-allele | CGCAGGCTGCTAATAAAATTTAGGT | AGAGTATCCATGTACAAGAGAGGAACA |
| loxP | GCTATACGAAGTTATTAGGTGATATCAGATCC | GGGCTTTCAGCTTAAAGTGGTTTAG |

**Table S2. Incubation time and amount of protein used in the urea assay for the determination of arginase activity in tissues**

| ***Organs*** | ***Protein amount in µg*** | ***Incubation time in min*** |
| --- | --- | --- |
| RBC lysates (human) | 100 | 60 |
| RBC ghosts (human) | 25 | 60 |
| RBC lysates (mouse) | 2000 | 60 |
| RBC ghosts (mouse) | 250 | 180 |
| Aorta | 200 | 60 |
| Heart | 200 | 300 |
| Lung | 200 | 40 |
| Liver | 2 | 10 |
| Kidney | 200 | 60 |
